# Poly‐d,l‐Lactic Acid Via Transdermal Microjet Drug Delivery for Treating Rosacea in Asian Patients

**DOI:** 10.1111/jocd.16556

**Published:** 2024-09-09

**Authors:** Suk Bae Seo, Jovian Wan, Jesper Thulesen, Arash Jalali, Massimo Vitale, Soo‐Bin Kim, Kyu‐Ho Yi

**Affiliations:** ^1^ SeoAhSong Dermatologic Clinic Seoul Korea; ^2^ Asia‐Pacific Aesthetic Academy Hong Kong Hong Kong; ^3^ Capitol Eye Clinic Copehagen Denmark; ^4^ One Clinic MD Vancouver Canada; ^5^ Private Practice Bologna Italy; ^6^ Division in Anatomy and Developmental Biology, Department of Oral Biology, Human Identification Research Institute, BK21 FOUR Project Yonsei University College of Dentistry Seoul Korea; ^7^ Maylin Clinic (Apgujeong) Seoul Korea

**Keywords:** needle‐free injector, poly‐d,l‐lactic acid, rosacea

## Abstract

**Background:**

Rosacea, a chronic inflammatory skin condition, is marked by enduring redness, visible blood vessels, and inflammatory eruptions in facial areas. Managing rosacea remains a persistent challenge for dermatologists, especially in cases unresponsive to conventional treatments. Injectable poly‐d,l‐lactic acid (PDLLA) has shown promise in treating erythema and telangiectasia associated with rosacea in addition to age‐related concerns. Employing Mirajet, a laser‐induced microjet system, for administering PDLLA is a novel and promising treatment for rosacea.

**Aims:**

We aimed to evaluate the efficacy and safety of injectable PDLLA delivered via a needle‐free microjet system for managing rosacea.

**Methods:**

Four Korean women with persistent and refractory rosacea received five monthly sessions of PDLLA needle‐free injections. Clinical assessments were conducted using the Clinician's Erythema Assessment and Patient's Self‐Assessment (PSA) at baseline, 4 weeks post‐treatment, and 22 weeks post‐final treatment. Adverse events were monitored throughout the study period.

**Results:**

At 4 weeks post‐treatment, both Clinician's Erythema Assessment and PSA scores indicated significant improvements in erythema that were sustained up to the 22‐week follow‐up. Patients reported high satisfaction with resolution of redness and improved skin texture. Mild swelling, redness, and petechiae were observed post‐treatment but resolved spontaneously. No product‐related adverse events were noted during the study period.

**Conclusion:**

Injectable PDLLA delivered via laser‐induced microjet injection demonstrated promising efficacy in improving rosacea symptoms and skin quality for up to 22 weeks without significant adverse effects. Larger randomized controlled trials are needed to confirm these findings and evaluate long‐term safety and sustainability of outcomes.

## 1 Introduction

Rosacea is a chronic, relapsing, inflammatory cutaneous condition that primarily affects the facial convexities, notably the cheeks, nose, forehead, and the chin. Its clinical manifestations encompass transient or persistent erythema, telangiectasia, and inflammatory lesions including papulo‐pustules and swelling. A defining feature of the condition is the persistent erythematous appearance of facial skin [1]. The etiology of rosacea is not fully understood and involves a complex interplay of factors including genetics, immune reactions, microorganisms, environmental triggers, and neurovascular dysregulation [2]. Genetic predisposition is evident in families with a history of the condition, and specific human leukocyte antigen loci have been identified in affected individuals. Although microorganisms like *Demodex* mites and *Helicobacter pylori* are thought to contribute, their exact roles are unclear [2, 3].

The literature suggests a multifaceted approach to treating rosacea, beginning with trigger avoidance and universal skincare recommendations such as pH‐balanced cleansers, high sun protection factor sunscreens, and gentle moisturizers [2, 3]. Cosmetic camouflage using green‐tinted products can help mask persistent erythema [4, 5]. Treatment selection is guided by individual symptoms, with a focus on inflammation reduction. Topical steroids should be avoided due to their potential for exacerbating symptoms [6]. Interventions targeting skin vasculature, such as brimonidine, oxymetazoline, or vascular laser therapy, may be beneficial for persistent redness and telangiectasias [7, 8, 9]. In conclusion, different treatment modalities for rosacea have demonstrated variability in outcomes due to the intricate pathogenesis of this condition. Therefore, tailoring treatment to individuals becomes essential, often requiring a combination of therapeutic modalities to comprehensively manage the diverse signs and symptoms of rosacea [10].

A study conducted by Seo et al. [11] evaluated the efficacy and safety of injectable poly‐d,l‐lactic acid (PDLLA) for skin rejuvenation. Following two or three treatment sessions, the results demonstrated improvements in various signs of skin aging, including fine wrinkles, skin texture, and irregular pigmentation. Interestingly, the study also revealed positive changes in erythema and telangiectasia, which are less commonly explored outcomes in research involving PDLLA interventions. These findings highlight a significant contribution to an understanding of the effects of PDLLA on skin conditions beyond traditional markers of aging.

Effective methods for delivering medication through the skin play a crucial role in dermatological procedures. In this study, we employed the laser‐induced microjet system, Mirajet (Mirajet, JSK Inc., South Korea), for administering PDLLA to manage rosacea. Mirajet functions on the principle of utilizing laser energy to deliver medication efficiently and uniformly to the targeted skin depth, without causing significant harm [12, 13, 14, 15]. Operating within a frequency range of 30–40 Hz, it generates rapid jet pressure, inducing vigorous vibrations and shocks within the dermal layer. These physical cues show potential in influencing cellular reactions, with repetitive tapping and micro‐tearing acknowledged as crucial stimuli for skin rejuvenation [14]. Among the variety of needle‐free injection devices available commercially, the laser‐induced microjet device is distinguished by its ability to achieve speeds of 40 Hz.

## 2 Materials and Methods

### 2.1 Patients

Four Korean women, aged between 43 and 52 years, with persistent and refractory rosacea were recruited for this study. [Correction added on 04 October 2024, after first online publication: In the preceding sentence, “47 and 53 years” has been changed to “43 and 52 years” in this version.] All participants sought treatment for rosacea and presented with Fitzpatrick skin type III. The research was conducted in accordance with the principles outlined in the Declaration of Helsinki. Given the nature of the study, an Institutional Review Board approval was deemed unnecessary and waived.

### 2.2 Inclusion and Exclusion Criteria

Inclusion criteria consisted of individuals diagnosed with rosacea, aged 18 years or older, and otherwise physically healthy. Exclusion criteria included individuals under 18 years of age, pregnant or lactating women, individuals currently receiving rosacea therapy, those who had received facial esthetic treatments including energy‐based devices within the past 3 months, and individuals with a history of hypersensitivity or allergic reactions to PDLLA.

### 2.3 Poly‐d,l‐Lactic Acid Preparation

A PDLLA 50 mg/hyaluronic acid (HA) 7.5 mg filler was dissolved in 10 mL of normal saline and vortexed for 2 h before use.

### 2.4 Treatment Protocol

The treatment regimen involved administration of PDLLA solution (Juvelook, VAIM Global, South Korea) via the microjet injection system (Mirajet, JSK Inc., South Korea). Patients underwent sessions once per month for a total of five sessions, with the injections administered by a single dermatologist until a total of 3 mL of diluted PDLLA (Juvelook, VAIM Inc., Seoul, Korea) was delivered. The injection technique employed a 200 𝜇m‐sized nozzle, operating at 20 Hz and delivering 14 000–15 000 pulses per session. Each pulse contained 0.2–0.3 𝜇L of the drug, comprising 16 mg of PDLLA in 3 mL of normal saline. The treatment end‐point was identified as the presence of papules with focal blanching and central pinpoint bleeding, and mild swelling at the injection sites.

To improve patient comfort, a topical anesthetic cream containing lidocaine and prilocaine was applied to the treatment area 30 min before the procedure. Additionally, a mixture of lidocaine and epinephrine was added to the solution to further reduce bleeding and discomfort and administered at low energy levels prior to the main treatment. Occasionally, minor bleeding occurred, and immediate pressure was applied to reduce the duration of bruising. Effective post‐procedural pressure treatment can minimize the duration of residual bruising. Minor bleeding was addressed by gently wiping the area with gauze soaked in a 10% dilution.

### 2.5 Clinical Assessment

Clinical digital photographs and three‐dimensional (3D) images were taken at baseline, before each procedural session, and during follow‐up appointments, which were scheduled 4 weeks and 22 weeks after the final treatment. Clinical photographs were taken with a digital camera and 3D images were acquired using a 3D camera (LifeViz Infinity; QuantifiCare, Biot, France). All images were captured under consistent positioning and lighting conditions in the photography room.

Patient improvement was evaluated at follow‐up appointments using the Clinician's Erythema Assessment (CEA), a five‐point grading scale for clinical assessment of the severity of facial erythema associated with rosacea, with confirmed high reliability [16]. This evaluation was completed by two independent dermatologists who were blind to the study details. Additionally, patient satisfaction with the outcome was self‐evaluated using the Patient's Self‐Assessment (PSA), a validated five‐point grading scale for patients to assess the severity of their facial erythema associated with rosacea [17].

### 2.6 Cases

#### 2.6.1 Case 1

A 47‐year‐old Korean woman with a history of rosacea presented with recurrent episodes of flushing and erythema. During her previous exacerbation of rosacea 6 months ago, she took minocycline (50 mg QD) orally for 4 months, resulting in the resolution of papules. At baseline assessment, both dermatologists graded her CEA as 2, indicative of mild erythema. Following 4 weeks of treatment, there was a marked improvement in erythema, with both dermatologists assigning a CEA score of 0, indicative of clear skin, which persisted up to the 22‐week follow‐up. Further, dermatologists observed improvements in pore size and skin texture. The PSA revealed a score of 0 at both the 4‐week and 22‐week follow‐ups, indicating the absence of unwanted redness as reported by the participant. Throughout the 22‐week follow‐up period, there were no notable adverse events or complications. The participant expressed satisfaction with the treatment outcomes, reporting increased confidence in her appearance.

#### 2.6.2 Case 2

**FIGURE 1:**
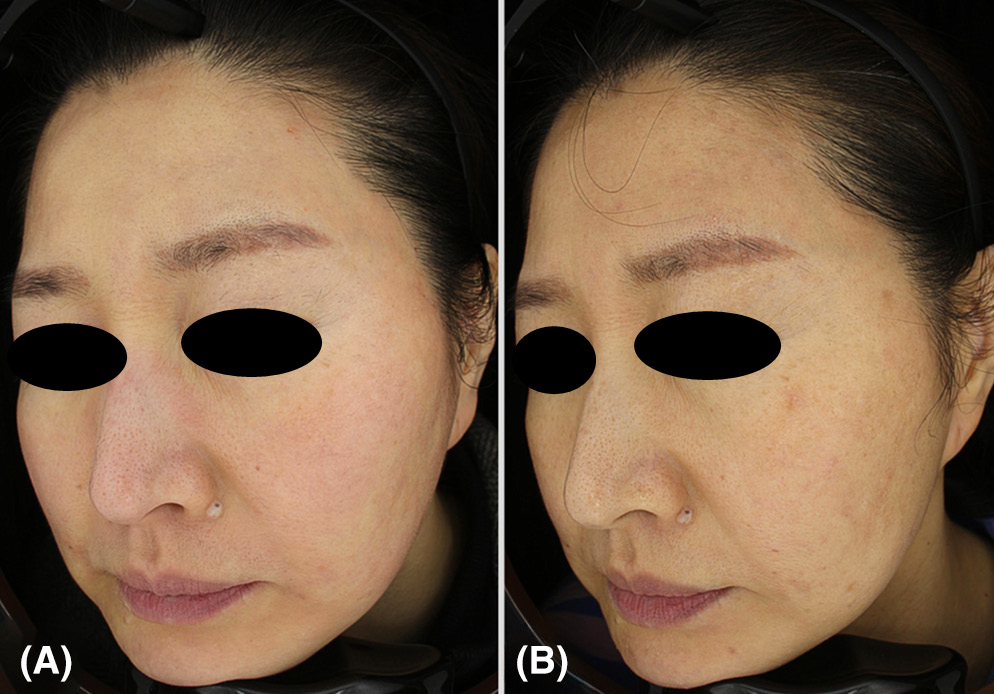
Three‐dimensional images of Case 2 (female, 49 years old), demonstrating the progression of rosacea following treatment. (A) Before treatment, showing moderate erythema (CEA Grade 3). (B) 22 weeks after final treatment (CEA Grade 2), demonstrating mild rosacea with improved pore size and skin texture.

A 49‐year‐old Korean woman presented with persistent rosacea and sought treatment. She had been experiencing rosacea for 7 years, which she had attributed to the birth of her first child and was not taking any medications for it. At baseline assessment, both dermatologists graded her CEA as 3, indicative of moderate erythema. Following 4 weeks of treatment, there was a marked improvement in erythema, with both dermatologists assigning a CEA score of 2, indicative of mild erythema, which persisted up to the 22‐week follow‐up. The PSA revealed a score of 0 at both the 4 and 22‐week follow‐ups, indicating the absence of unwanted redness as reported by the participant. Throughout the 22‐week follow‐up period, there were no notable adverse events or complications (Figure 1). The participant expressed satisfaction with the treatment outcomes, noting that her skin looked and felt smoother.

#### 2.6.3 Case 3

**FIGURE 2:**
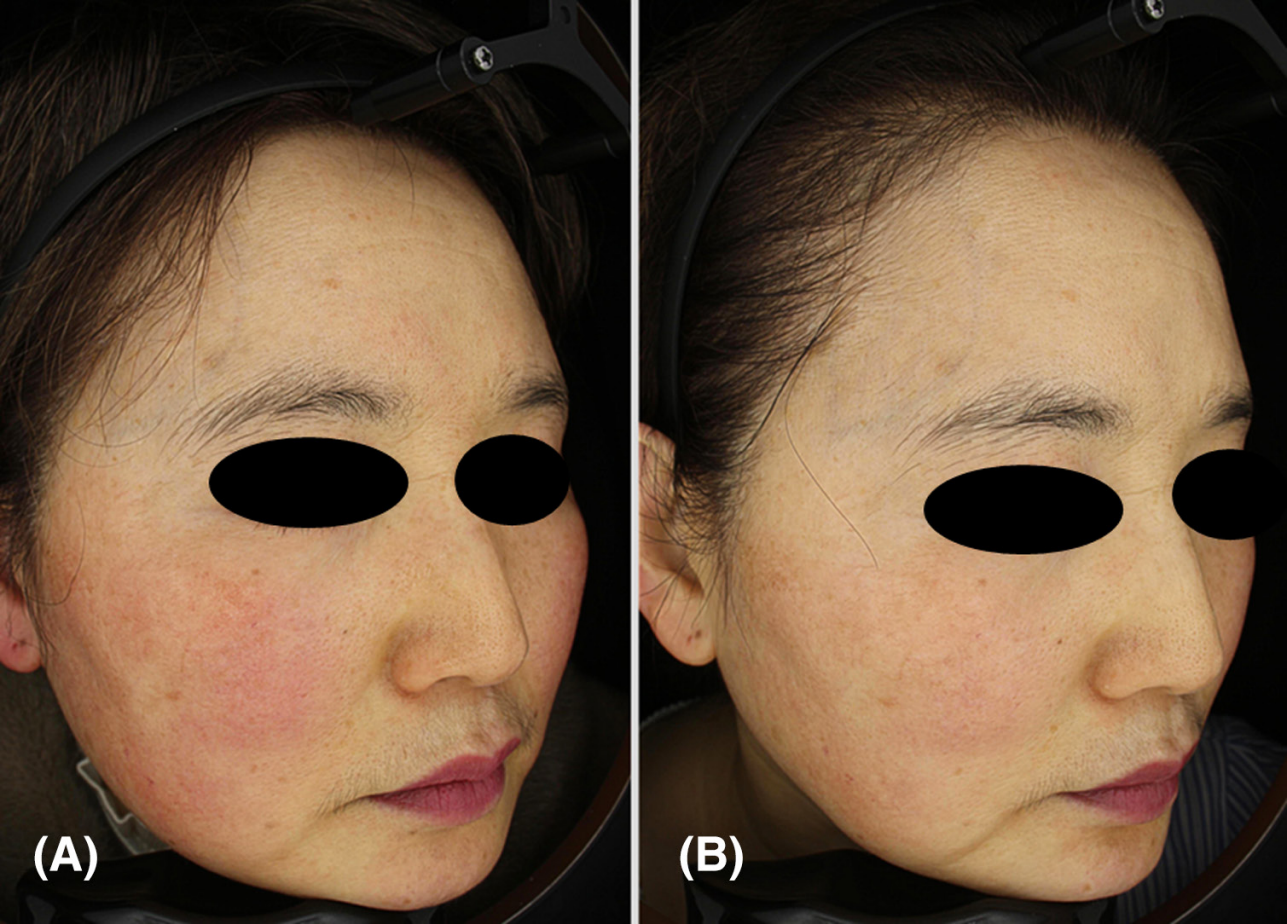
Three‐dimensional images of Case 4 (female, 52 years old), demonstrating the progression of rosacea following treatment. (A) Before treatment, showing severe erythema (CEA Grade 4). (B) 22 weeks after the final treatment (CEA Grade 1), demonstrating almost clear skin with improved pore size and skin texture. [Correction added on 04 October 2024, after first online publication: Figure 2 caption has been updated in this version.]

A 52‐year‐old Korean woman presented with refractory rosacea, having previously tried minocycline courses and laser treatment with no improvement (Figure 2). At baseline assessment, both dermatologists graded her CEA as 3, indicative of moderate erythema. Following 4 weeks of treatment, there was a marked improvement in erythema, with both dermatologists assigning a CEA score of 0, indicative of clear skin, which persisted up to the 22‐week follow‐up. The PSA revealed a score of 0 at both the 4‐week and 22‐week follow‐ups, indicating the absence of unwanted redness as reported by the participant. Throughout the 22‐week follow‐up period, there were no notable adverse events or complications. The participant expressed great satisfaction with the results.

#### 2.6.4 Case 4

**FIGURE 3:**
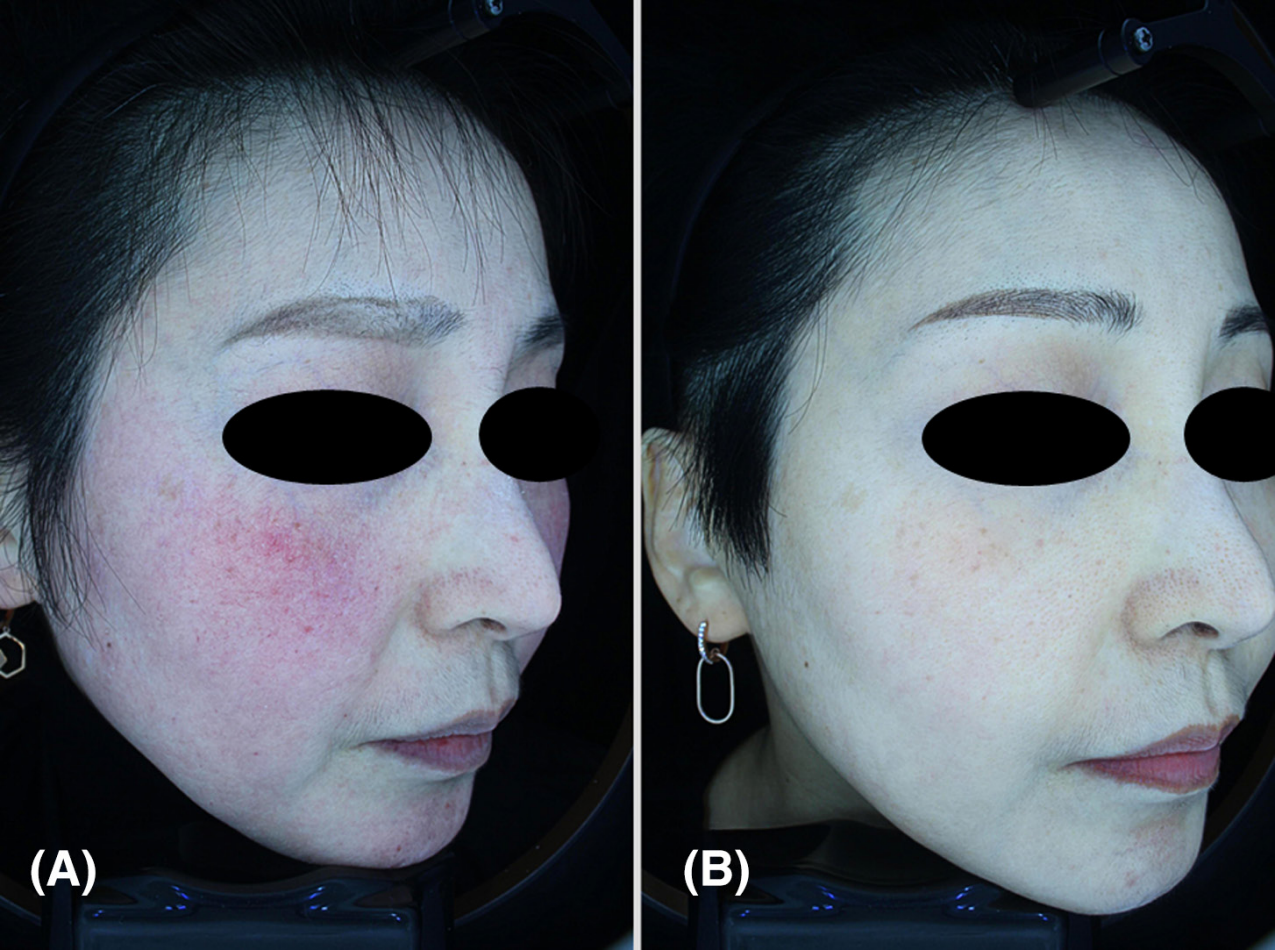
Close‐up three‐dimensional images of the right cheek of Case 4 (female, 43 years old), demonstrating marked improvement in severe erythema following treatment with poly‐d,l‐lactic acid via a needle‐free microjet. The images depict the condition before (A) and 22 weeks after the final treatment (B).

A 43‐year‐old woman presented with a 5 year history of rosacea, which had been conservatively managed. She also had a family history of rosacea (Figure 3). At baseline assessment, both dermatologists graded her CEA as 4, indicating severe erythema. After 4 weeks of treatment, there was a notable improvement in erythema, with both dermatologists assigning a CEA score of 1, indicating almost clear skin, which persisted up to the 22‐week follow‐up. The PSA also revealed a score of 0 at both the 4‐week and 22‐week follow‐ups, indicating that the participant had self‐reported an absence of unwanted redness (Figure 3). Throughout the 22‐week follow‐up period, there were no notable adverse events or complications. The participant noted improvements to pore size and skin texture.

## 3 Results

At the 4‐week post‐final treatment follow‐up, assessments from both the CEA and PSA showed positive results, with scores remaining consistent at the 22‐week follow‐up. Patients expressed high satisfaction with their rosacea improvement and noted enhancements in skin texture, including pore size, and smoothness (Table 1). During the procedure, patients reported slight discomfort, with pain scores ranging from 1 to 2 on a scale of 0–5. Following each treatment, patients experienced mild swelling and increased redness, persisting for approximately 36 hours. Mild petechiae were observed in all patients but resolved on their own within 72 hours. No adverse events related to the product, such as nodule formation or local inflammation, were noted during the 22‐week follow‐up period.

**TABLE 1:** A summary of the assessment scores used in the study.

Participants	CEA by Physician 1 before treatment	CEA by Physician 2 before treatment	CEA by Physician 1 at 4 weeks post‐final treatment	CEA by Physician 1 at 22 weeks post‐final treatment	CEA by Physician 2 at 4 weeks post‐final treatment	CEA by Physician 2 at 22 weeks post‐final treatment	Patient Self‐Evaluation Score at 4 weeks post‐final treatment	Patient Self‐Evaluation Score at 22 weeks post‐final treatment
1	2	2	0	0	0	0	0	0
2	3	3	2	2	2	2	1	1
3	3	3	1	0	0	0	1	0
4	4	4	1	1	1	1	0	0

*Note:* The Clinician's Erythema Assessment (CEA) scores were evaluated independently by two blinded physicians 4 and 22 weeks after the final treatment, reflecting the overall improvement in erythema. Patient satisfaction scores, obtained through self‐reporting using the Patient's Self‐Assessment (PSA), indicate the level of satisfaction with the facial erythema associated with rosacea.

## 4 Discussion

Treating rosacea poses ongoing challenges for dermatologists, particularly in cases that are resistant to conventional therapies. The mechanisms underlying rosacea development are still not fully comprehended [18]. This is the first study to investigate the efficacy and safety of using laser‐induced microjet injectors to administer PDLLA, tailored for the treatment of patients with rosacea.

In the quest for novel treatments for rosacea, botulinum toxin has emerged as a potential solution. It has demonstrated efficacy in addressing persistent erythema and flushing associated with rosacea by inhibiting mast cell degranulation and acetylcholine release, while also modulating substance P, calcitonin gene‐related peptide, and vasoactive intestinal peptide [19]. According to various studies, botulinum toxin can alleviate symptoms of rosacea, such as flushing and erythema, for a duration of at least 8 weeks, with its effects typically lasting between 3 and 4 months [20]. In the case report by Yu et al. [21], botulinum toxin was administered to a female patient with rosacea, resulting in temporary improvement. However, 1 month after the injection, the erythema returned, following a 1‐month absence of symptoms. Subsequently, the patient received poly‐l‐lactic acid (PLLA) via injection with a mesogun. Notably, 1 week after the injection, the patient experienced a recurrence of flushing and redness, which gradually subsided after 4 weeks without further treatment [21].

**FIGURE 4:**
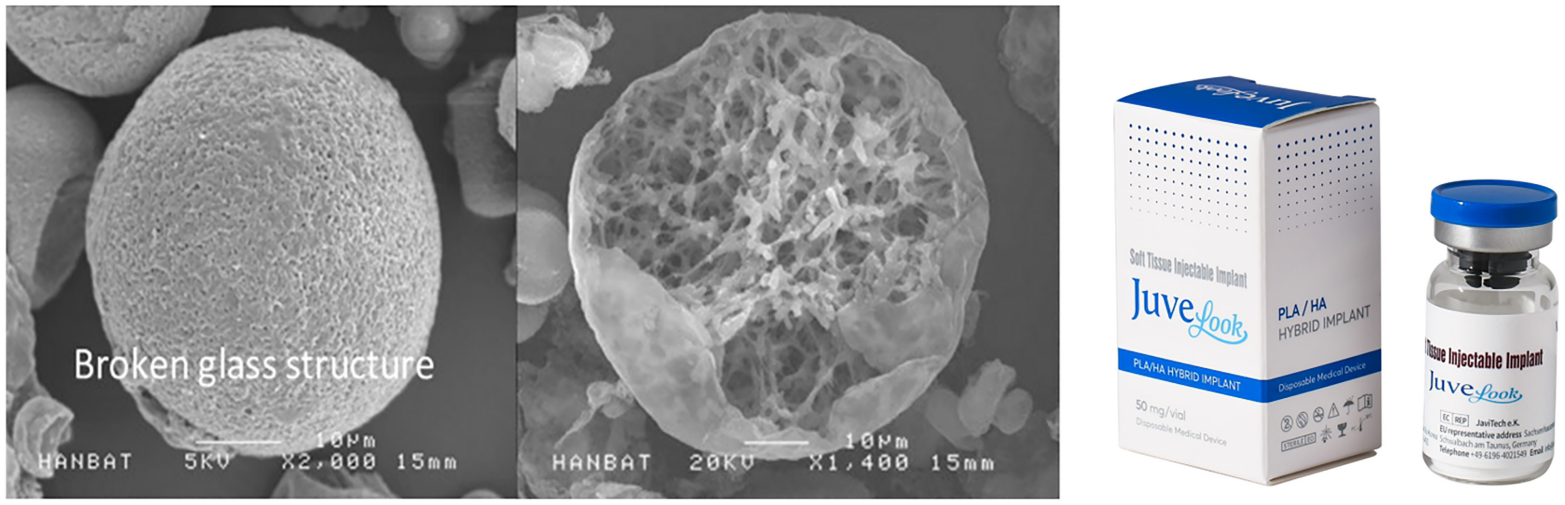
SEM image of the PDLLA (Juvelook, VAIM Inc., Korea). The PDLLA microparticles are spherical in shape with multiple pores on the surface, and have a diameter of 30–70 μm. The microparticle size of both PDLLA monomers make them small enough to pass through an injection needle, but large enough to protect them from phagocytosis. The PLAs may have adverse effect of nodule formation, however, PDLLA produced by VAIM Inc. has foamy structures which can be degrades using energy‐based devices.

Injectable PDLLA and PLLA are both biocompatible, biodegradable, and biostimulatory substances used in esthetic procedures. However, they differ in composition and characteristics. PDLLA is amorphous with an irregular chain structure, while PLLA is hemi‐crystalline with a regular chain structure. PDLLA degrades faster and has a lower glass transition temperature, melting temperature, and tensile strength compared to PLLA (Figure 4). Additionally, PDLLA microparticles are spherical with multiple pores, whereas PLLA microparticles are irregularly shaped. Despite these differences, both PDLLA and PLLA must be reconstituted with sterile water before injection, and they both utilize carboxymethyl cellulose for reconstitution [22]. Regarding rosacea treatment, the differences between PDLLA and PLLA may impact their effectiveness and duration of action. However, further comprehensive studies with objective assessments are necessary to evaluate the efficacy of both injectable materials in managing rosacea symptoms.

Vascular endothelial growth factor (VEGF) and collagen stimulation induced by PDLLA significantly contribute to reducing flushing in rosacea patients by reinforcing the basement membrane and enhancing vascular stability. VEGF plays a crucial role in promoting angiogenesis, which not only improves blood vessel function but also facilitates overall skin rejuvenation. This enhanced vascular function helps mitigate the erratic blood flow often seen in rosacea, thereby reducing the occurrence of flushing. Concurrently, the increased collagen synthesis fortifies the structural integrity of the skin, providing a more robust barrier against environmental and physiological triggers of rosacea. The combined effects of improved angiogenesis and strengthened collagen networks make the skin more resilient and less prone to the characteristic redness and irritation associated with rosacea [23].

This study has several limitations that warrant consideration in future research. First, the small sample size was inadequate to achieve statistical power. Additionally, the study cohort consisted exclusively of Korean participants, all of whom were women and had Fitzpatrick skin type III. Focusing solely on this demographic may introduce potential outcome variations when generalizing findings to populations with diverse skin phototypes and ethnicities, as previous research has highlighted differences in skin properties across ethnic groups [24]. Women are more commonly affected by rosacea than men; however, in order to ensure a comprehensive understanding of the effectiveness of the treatment across the sexes, future studies should include men as well [8]. Further, all patients received treatment from the same practitioner, which may introduce variability in visible outcomes due to differences in practitioner experience and techniques.

Our study included patients with varying degrees of erythema, from mild to severe, as well as those with refractory and persistent melasma, reflecting the broad spectrum of conditions we encounter in clinical practice. While we observed that mild rosacea often responds more consistently to treatment in clinical practice, this was not a focus of our study, nor did our findings specifically suggest this. Given the complexity and challenges in treating persistent and severe rosacea, future research should priortize these more difficult cases. This approach could lead to a better understanding of treatment efficacy across a broader spectrum of rosacea severity and contribute to the development of more targeted strategies for managing these more challenging forms of the condition.

The primary visible clinical changes noted in this study, involving the application of PDLLA delivered by needle‐free microjet injection, included a sustained improvement in rosacea for 22 weeks. An overall improvement in skin quality was also noted. Further research, especially employing randomized controlled trials, is needed to validate these findings and assess the longer‐term safety and sustainability of outcomes.

## Author Contributions

Conceptualization: Suk Bae Seo, Kyu‐Ho Yi. Writing – Original Draft Preparation: Jovian Wan, Soo Bin Kim. Writing – Review & Editing: Suk Bae Seo, Jovian, Wan, Jesper Thulesen, Massimo Vitale. Visualization: Soo‐Bin Kim, Arash Jalali. Supervision: Kyu‐Ho Yi. All authors have reviewed and approved the article for submission.

## Ethics Statement

This study was conducted in compliance with the principles set forth in the Declaration of Helsinki.

## Conflicts of Interest

The authors declare no conflicts of interest.

## Data Availability

The data that support the findings of this study are available from the corresponding author upon reasonable request.
